# Thermal Diffusion Films with In-Plane Anisotropy by Aligning Carbon Fibers in a Cellulose Nanofiber Matrix

**DOI:** 10.1021/acsami.2c09332

**Published:** 2022-07-20

**Authors:** Kojiro Uetani, Kosuke Takahashi, Rikuya Watanabe, Shota Tsuneyasu, Toshifumi Satoh

**Affiliations:** † SANKEN (The Institute of Scientific and Industrial Research), Osaka University, Mihogaoka 8-1, Ibaraki-shi, Osaka 567-0047, Japan; ‡ Graduate School of Engineering, Osaka University, Mihogaoka 8-1, Ibaraki-shi, Osaka 567-0047, Japan; § Department of Media Engineering, Graduate School of Engineering, Tokyo Polytechnic University, 1583 Iiyama, Atsugi, Kanagawa 243-0297, Japan; ∥ Department of Electrical and Electronic Engineering, National Institute of Technology, Oita College, 1666 Maki, Oita 870-0152, Japan

**Keywords:** in-plane anisotropy, aligned carbon fibers, thermal interference, carbon-fiber reuse, powder electroluminescent device

## Abstract

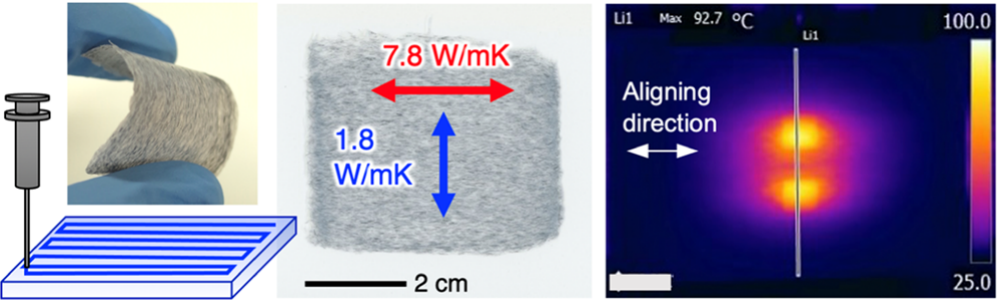

For highly efficient heat dissipation of thin electronic devices, development of film materials that exhibit high thermal conductivity in the in-plane direction is desired. In particular, it is important to develop thermally conductive films with large in-plane anisotropy to prevent thermal interference between heat sources in close proximity and to cool in other directions by diffusion. In this study, we developed flexible composite films composed of a uniaxially aligned carbon-fiber filler within a cellulose nanofiber matrix through liquid-phase three-dimensional patterning. The film exhibited a high in-plane thermal conductivity anisotropy of 433%, with combined properties of a thermal conductivity of 7.8 W/mK in the aligned direction and a thermal conductivity of 1.8 W/mK in the in-plane orthogonal direction. This remarkable thermal conductivity and in-plane anisotropy showed the ability to significantly cool powder electroluminescent devices formed on the composite film and also to cool two heat sources in close proximity without thermal interference. In addition, the carbon-fiber filler could be extracted from the composite films by heat treatment at 450 °C and reused as a thermally conductive material.

## 1 Introduction

Recently, thin and flexible paper-like electronic devices have been developed.^1−3^ As their performance improves, the problem of heat exhaustion will become more serious. Because thin devices cannot be equipped with conventional bulky heat sinks, thermal diffusion to the substrate and convective heat dissipation by kirigami processing^4^ are considered to be promising for cooling the devices. Thermal diffusion through the substrate in contact with the heat-generating device is the most important method for heat dissipation, and thus it is essential to improve the thermal conductivity of the substrate material in the in-plane direction.^5^ However, the problem of the thermal interference between neighboring devices could arise with improvement of the thermal conductivity of the substrate. For a substrate on which multiple devices are mounted in high density, it is necessary to control the direction of thermal diffusion and find an effective heat-removal path while thermally insulating between the devices. Therefore, the development of substrate films with high in-plane thermal conductivity anisotropy remains an important target.

Polymer-based films are widely used as substrate materials for electronic devices, which require flexibility and electrical insulation, and many polymer composites with large amounts of thermally conductive fillers have been suggested to enhance the thermal conductivity. The improvement in the thermal conductivity is highly dependent on the shape of the filler. Particulate fillers, such as alumina, do not show thermal conductivity anisotropy as a whole material, and they need to be added in large quantities to improve the thermal conductivity.^6^ Plate-like fillers, such as alumina platelets and boron nitride, are readily in-plane oriented in the film and have a high thermal conductivity in the in-plane direction.^7−9^ Therefore, the heat-transfer anisotropy between the thickness direction and in-plane direction could be large. However, there have been few reports on the development of heat-transfer anisotropy within the film plane to prevent thermal interference between neighboring devices. Fibrous fillers, such as copper nanowires and carbon fibers (CFs), are highly effective in improving the thermal conductivity of polymer composites when they are added in small amounts owing to their large structural anisotropy.^10,11^ Furthermore, the in-plane alignment of the fiber filler is expected to develop in-plane anisotropy in two-dimensional (2D) materials.

The candidates for matrix polymers include petroleum-based plastics, rubber, glass, and biopolymers. Among these materials, the film of cellulose nanofibers (CNFs) extracted from the mantle of ascidian has been reported to show higher thermal conductivity (∼2.5 W/mK) than other conventional polymers due to their thick crystallines with the extended chain crystal structure,^12^ and characterization of their thermal conduction and the use of CNFs as a heat-dissipation material have been reported.^4,13−16^ As indicated by the ability of pencils to write characters on paper, cellulose has a high affinity for carbon materials, and it is expected to composite well with CF fillers. In addition, CNF can be easily handled in an aqueous system, and it has the advantage of self-agglomeration to form a film (paper) upon drying. Furthermore, in-plane aligned films can be constructed from aqueous dispersed CNFs by liquid-phase three-dimensional (3D) patterning.^17^ It has also been reported that a similar 3D patterning process can be used for CF-containing polymers to form lightweight composite materials with aligned CF-filler fibers.^18,19^ We believe that 3D patterning of an aqueous CNF and CF mixture could form a highly anisotropic thermally conductive film with aligned CFs.

Another advantage of using CFs and CNFs is that waste can be reduced by recycling the CF filler, thus decreasing the environmental impact.^20,21^ Although there are reported cases of recycling and reuse of CFs as polymer reinforcement materials,^22−26^ little consideration has been given to recycling and reuse of CF fillers toward thermally conductive composites. Because CF has a higher pyrolysis temperature than cellulose, it is expected that CF can be isolated intact by heat treatment of a CF and CNF composite at an intermediate temperature between the pyrolysis temperatures of the two materials. In other words, it is expected that the filler of the CF/CNF thermally conductive composite can be recovered and reused as a filler.

In this study, uniaxially aligned CF/CNF composite films were synthesized by liquid 3D patterning of an aqueous suspension of CFs and ascidian-derived CNFs, and thermal conductivity anisotropy in the film plane was demonstrated. A top-emission-type powder electroluminescent (EL) device was mounted on the film, and its heat-dissipation performance was tested. Two proximate heat sources were pseudo-formed, and the heat insulation between the two heat sources and heat dissipation in different directions were simultaneously evaluated. In addition, the CF was extracted from the composite film by heat treatment at a specific temperature, and its reusability as a thermally conductive filler was investigated.

## 2 Experimental Section

### 2.1 CNF Preparation

The mantle of the ascidian (*Halocynthia roretzi*) was cut into ∼1 cm × 1 cm pieces, and then ∼50 g was immersed in 1.5 L of water at 80–90 °C. NaClO_2_ was added to make the concentration 1 wt %, and bleaching was performed with stirring for 1 h under acetic acidic conditions. This treatment was continuously repeated for a total of 3 h until the product became white. The product was washed with deionized water and filtered to obtain white ascidian-derived cellulose. Undried ascidian-derived cellulose (5 g in dry weight) was suspended in 500 mL of water, 0.5 g of NaBr, 0.08 g of 2,2,6,6-tetramethylpiperidine-1-oxyl (TEMPO), and 48.6 mmol of aqueous NaClO solution (∼1.6 M) were added, and the solution was stirred. The reaction was carried out for 6 h using 0.1 M NaOH solution to maintain the pH of the solution at 10–11. After treatment, the product was washed with deionized water, and ascidian-derived TEMPO-oxidized cellulose with a surface carboxyl group content of 0.53 mmol/g was obtained. The TEMPO-oxidized cellulose was suspended in deionized water at a concentration of 0.5 wt % and stirred with a high-speed blender for 30 min to produce ascidian-derived TEMPO-oxidized CNFs.

### 2.2 CF/CNF Composite Preparation

Long fibers of pitch-based CFs (Dialead K13D2U, Mitsubishi Chemical Corporation, Tokyo, Japan) were cut into ∼1 mm lengths by Nippon Polymer Sangyo Co., Ltd. (Osaka, Japan). Heat treatment was performed with an electric furnace (KDF-75, Denken-Highdental Co., Ltd., Kyoto, Japan) at 400 °C for 1 h in an air atmosphere to remove the sizing agent coated on the CF surface. The temperature increase and decrease rates were set to 10 and −2.5 °C/min, respectively. Thermogravimetric analysis (TGA, Q50, TA Instruments Japan, Tokyo, Japan) was performed to estimate the weight of the removed sizing agent.

Aligned CF/CNF composite films were prepared from the CNF suspension mixed with 10 wt % CF with respect to the dry CNF content by liquid-phase 3D patterning using a combination of a three-axis robot (BS-101005-1674, COMS Co., Ltd., Hyogo, Japan) and a syringe pump (PHD ULTRA 70-3005, Harvard Apparatus, Holliston, MA) considering a previous study.^17^ A 200 μL pipette tip (epT.I.P.S., Eppendorf Japan, Tokyo, Japan) was used as the discharging needle. The patterning speed was set to 34 mm/s, and the optimal discharging rate was applied. A mixture of acetone (140 mL) and 1 M HCl (60 mL) was used as a coagulant to suppress air bubbles and ensure precise patterning. After finishing the patterning, the coagulant was gently removed with an electric pipettor, and the gel formed after patterning was dried in an oven at 55 °C for 2 days to produce unidirectionally aligned films with an apparent thickness of 75–110 μm, which are called Aligned-CF10.

CF/CNF aqueous suspensions were prepared by adding CF to CNF suspensions at 0, 5, 10, 15, and 20 wt % of the dry CNF weight. The mixed suspensions were stirred with a magnetic stirrer for 3–4 h to dissociate the bundled CFs. The suspensions were then vacuum-filtered through a membrane filter (pore size 0.1 μm, Advantec Toyo Kaisha, Ltd., Tokyo, Japan) to form wet mats. After covering the tops of the wet mats with a new membrane filter, the mats were sandwiched between metal meshes (300 mesh) with paper towels on both sides. The mats were then dried in a hot-press machine at 110 °C for 20 min to produce randomly aligned CF/CNF films with an apparent thickness of 70–150 μm, which are called Random-CF*x*, where *x* is the weight percentage of CF in the composite.

### 2.3 Characterization of the Composite Films

The thermal diffusivity of the composite films was measured in the thickness direction and in any in-plane direction with a TA33 thermowave analyzer (Bethel Co., Ltd., Ibaraki, Japan) based on the laser spot periodic heating radiation thermometry method. A laser whose intensity is sinusoidally modulated is irradiated onto the surface of the film sample, and the temperature response of the back surface is read by a radiation thermometer. When the backside detection position is directly behind the laser irradiation spot, the thermal diffusivity in the thickness direction is measured; when the backside detection positions are far from the laser spot, the in-plane thermal diffusivity in the direction from the laser spot to the detection position is measured.^12^ Both sides of the sample were blackened with graphite spray (FC-142, Fine Chemical Japan Co., Ltd., Tokyo, Japan) to prevent laser penetration and maintain high and constant emissivity. The thermal conductivity was calculated by multiplying the thermal diffusivity by the bulk density of the film and specific heat capacity. The specific heat capacity of the CF/CNF composite film (*C*_p_) with a CF content of *x* wt % was calculated from the specific heat capacities of CFs and CNFs (*C*_p-CF_ and *C*_p-CNF_, respectively) measured with a differential scanning calorimeter (Thermo Plus Evo2 8230, Rigaku, Tokyo, Japan) by *C*_p_ = *xC*_p-CF_ + (1 – *x*)*C*_p-CNF_.

The optical images of the composite-film surfaces were taken with a flatbed scanner (GT-X830, Seiko Epson Corp., Nagano, Japan) at 1200 dpi. The angle distribution of CF in the composite films was measured using ImageJ 1.53a (National Institutes of Health, Bethesda, MD). The surface resistivity of the CF/CNF films was measured with a Hiresta-UX resistivity meter (MCP-HT800, Mitsubishi Chemical Analytech Co., Ltd., Kanagawa, Japan) using a UR-SS-type probe (MCP-HTP15). Measurements were performed for both the air side and filter side during film formation. The surface roughness of the CF/CNF films was measured with a 3D laser scanning microscope (OLS5100, Olympus Corp., Tokyo, Japan). The root-mean-square heights (*S*_q_) of both the air and filter sides of the films were calculated.

### 2.4 Preparation of the Top-Emission-Type EL Devices

Top-emission-type powder EL devices were fabricated considering the fabrication method for bottom-emission-type powder EL devices reported in our previous studies.^27,28^ As the back electrode, Ag paste (MP-603S, Mino Group Co., Ltd., Gifu, Japan) was coated on the CF/CNF films by an automatic screen-printing machine (TU2020-C, Seritech Co., Ltd., Osaka, Japan) and dried at 80 °C for 45 min. Next, a high-dielectric binder was prepared by mixing cyclohexanone (037-05096, Fujifilm Wako Pure Chemical Co.) and cyanoethyl cellulose-type high-dielectric polymers (CR-V, Shin-Etsu Chemical Co., Ltd., Tokyo, Japan) at a weight ratio of 7:3. The dielectric paste was prepared by mixing BaTiO_3_ (620-07545, Kishida Chemical Co., Ltd., Osaka, Japan) with the high-dielectric binder at a weight ratio of 6:4. This dielectric paste was coated on the back electrode by the screen-printing method and dried at 80 °C for 6 min to form the dielectric layer. A phosphor paste was prepared by mixing a commercial ZnS-type phosphor (GG45, Osram Sylvania, Wilmington, MA) with the binder at a weight ratio of 6:4. This phosphor paste was coated on the dielectric layer by the screen-printing method and dried at 80 °C for 6 min, forming a phosphor layer. To form a transparent electrode, poly(2,3-dihydrothieno-1,4-dioxin)–poly(styrenesulfonate) (768650, Sigma-Aldrich Co., LLC, Tokyo, Japan) was coated on top of the phosphor layer by the screen-printing method and dried at 80 °C for 10 min. Through the above processes, top-emission-type powder EL devices with an emission area of 1 cm × 1 cm were fabricated. An EL measurement system (SX-1152, Iwatsu Electric Co., Ltd., Tokyo, Japan) was used to evaluate the current and luminance of the powder EL devices.

### 2.5 Heat-Dissipation Test

The heat-dissipation tests were conducted in an indoor environment of 25 ± 0.5 °C and 15% relative humidity. In the heat-dissipation tests of the EL devices, the dimensions of the EL substrate were standardized to 4 cm × 5 cm, and the temperature distribution behind the emitting surface was observed with a thermal camera (ETS320, FLIR Systems, Inc., Wilsonville, OR) when an alternating-current (AC) voltage of 170 V at 1.2 kHz was applied to the device. The EL device was held on the acrylic hollow stage by fixing the electrode tape so that the EL substrate would not come into contact with the other component, as much as possible.

In the heat-dissipation test using two proximate heat sources, two 5 mm × 5 mm blackened areas were first formed in the center of the film with dimensions of 35 mm × 45 mm. For Aligned-CF10, the two blackened areas were placed perpendicular to the CF-aligning direction. The film was placed on a holding jig with the blackened surface facing the ground, and a solar simulator (HAL-320W, Asahi Spectra Co., Ltd., Tokyo, Japan) was used to irradiate white light from underneath the film at an irradiance of ∼0.5 W/cm^2^ measured by a PM160T power meter (Thorlabs, Inc., Newton, NJ). The blackened area generates heat through light absorption and functions as a pseudo heat source. After maintaining the light irradiation for ∼5 min, the steady-state temperature distribution was observed by an ETS320 thermal camera.

### 2.6 CF Reuse Test

The CF/CNF composite film was subjected to heat treatment using a KDF-75 electric furnace to extract the CF. The temperature increase and decrease rates were set to 10 and −2.5 °C/min, respectively. The Random-CF10 film was cut into ∼5 mm squares and heated at 450 °C in the electric furnace. The weight of the obtained combustion residue (the residue of the first combustion is called CR1) was measured. CR1 was then mixed with a new CNF suspension as a filler at 10 wt % of the dry CNF weight to make new random CF/CNF films, which are called CR1-10%. This process of heat treatment and formation of a new film is regarded as a single CF reuse cycle, and another cycle was performed to obtain CR2 to form the films CR2-10%. The thermal diffusivity of the composite films after each cycle was measured by a TA33 thermowave analyzer. TGA measurements were also performed with the same temperature program as in the electric furnace to investigate the combustion behavior.

## 3 Results and Discussion

**Figure 1:**
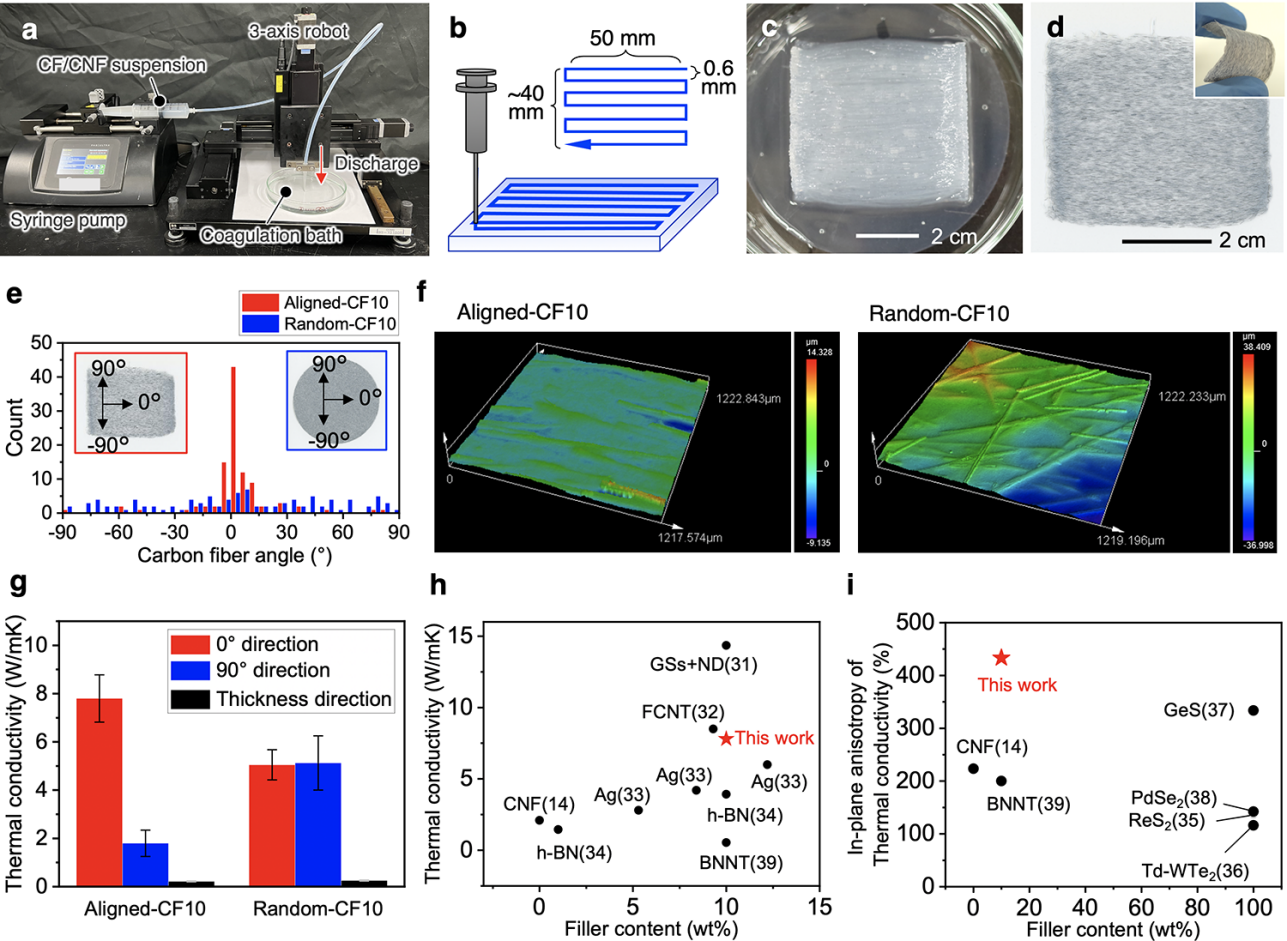
Aligned CF/CNF composite films. (a) Liquid-phase 3D patterning setup and (b) patterning program used. (c) Appearance of the patterned gel when the coagulum was gently removed after patterning. (d) Aligned-CF10 film is formed by drying the patterned gel. The insert shows its flexibility. (e) CF-alignment-angle distributions in the Aligned-CF10 and Random-CF10 films. (f) Height profile on the filter side of each film. (g) Thermal conductivities of the CF/CNF films in each direction. (h) In-plane thermal conductivity and (i) in-plane anisotropy of the thermal conductivity versus the filler content for various 2D materials with the following abbreviations; GSs+ND, graphene sheets and nanodiamonds; FCNT, fluorinated carbon nanotube; h-BN, hexagonal boron nitride; Td-WTe_2_, Td-polytype tungsten ditelluride; and BNNT, boron nitride nanotubes. The numbers in parentheses indicate the reference numbers.

The procured 1-mm-long CFs were purified by heating them at 400 °C for 1 h to remove the surface sizing agent (∼1.7 wt %, Figure S1). The purified CF did not sink in water by itself, but it was suspended in water in the presence of CNFs. A CNF suspension mixed with 10 wt % CFs with respect to the dry CNF content was discharged from a thin needle and unidirectionally aligned (Figure 1a). The reason for selecting 10 wt % CF loading in the aligned composite film is that preliminary experiments have shown that CFs can be patterned most smoothly without clogging the dispensing needle or causing uneven dispersion, and the concentration is directly comparable to that of the random film. Acidic aqueous solution (i.e., hydrochloric acid) is known to be an effective coagulation bath for gelation of TEMPO-oxidized CNFs.^29^ However, the specific gravity of hydrochloric acid was too high and the patterning gel floated. Thus, it was mixed with acetone, which also has a gelation effect on CNFs.^30^ To suppress bubble generation during solvent replacement, the coagulation solution was optimized by mixing 140 mL of acetone with 60 mL of 1 M HCl to achieve stable patterning.

The patterned gel maintained its 3D shape after the coagulant was removed from the Petri dish (Figure 1c). After drying, it became a film (Aligned-CF10, Figure 1d), and the alignment structure of the CF was fixed. The film was flexible, and even if it was deformed, the CF hardly fell out of the composite film. The alignment of the CFs in the film was evaluated (Figure 1e). Most of the CFs were located in the patterning direction (defined as 0°), and ∼72% of the CFs were in the angle range of ±10°. Liquid-phase 3D patterning allowed the CFs to be aligned almost as intended. In contrast, the CF angles of Random-CF10 were widely and uniformly distributed from −90 to 90° when 0° was set as an arbitrary direction for convenience, showing that Random-CF10 had a distinctly different structure to Aligned-CF10.

The difference in the orientation structures was also evident from 3D images obtained by laser microscopy (Figure 1f). Although the surface roughness of the air side (during drying) of Aligned-CF10 and Random-CF10 was not significantly different, the roughness of the filter side of Aligned-CF10 (*S*_q_ = 1.537 μm) was significantly smaller than that of Random-CF10 (*S*_q_ = 4.588 μm). CF alignment appeared to reduce intersection of the CFs and thus the surface roughness.

To investigate the in-plane thermal conductivity anisotropy of Aligned-CF10, the thermal diffusivities in the specific 0 and 90° directions in the plane were independently measured by laser-spot periodic heating radiation thermometry (Figure 1g). For Aligned-CF10, the thermal conductivity was 7.8 W/mK in the 0° direction (*k*_0°_), 1.8 W/mK in the 90° direction (*k*_90°_), and 0.21 W/mK in the thickness direction, showing 433% thermal conductivity anisotropy in the film plane, which is calculated by *k*_0°_/*k*_90°_. In contrast, the thermal conductivity of Random-CF10 was ∼5.1 W/mK in both the 0 and 90° directions, and no in-plane anisotropy was observed. Therefore, clear thermal conductivity anisotropy was obtained by CF alignment.

Among reported 2D film materials, Aligned-CF10 achieved relatively high thermal conductivity for a similar filler content (Figure 1h). Composite-film materials with a combination of graphene sheets and nanodiamonds^31^ and fluorinated carbon nanotubes^32^ exhibit higher in-plane thermal conductivity than Aligned-CF10 at a filler content of ∼10 wt %. However, these materials are in-plane isotropic and do not exhibit in-plane anisotropic thermal conduction.^31−34^ Among the 2D materials showing in-plane thermal conductivity anisotropy in Figure 1i, Aligned-CF10 exhibits the highest level of thermal conductivity anisotropy (433%). The materials with a filler content of 100 wt % are 2D sheet-like inorganic crystalline materials.^35−38^ We cited the thermal conductivity and its anisotropy of the material with unidirectional alignment of BNNT in a poly(vinyl alcohol) matrix.^39^ Using conventional components of polymer composites, it is difficult to arrange the filler anisotropically in the film plane, and there are limited methods to evaluate the thermal conductivity of each direction in the plane independently. We have successfully fabricated a thermally conductive film with in-plane anisotropy by suitable material selection and bottom-up structuring by 3D patterning, and we clearly confirmed the in-plane anisotropy by direction-specific thermal diffusivity measurements by laser spot periodic heating radiation thermometry.

**Figure 2:**
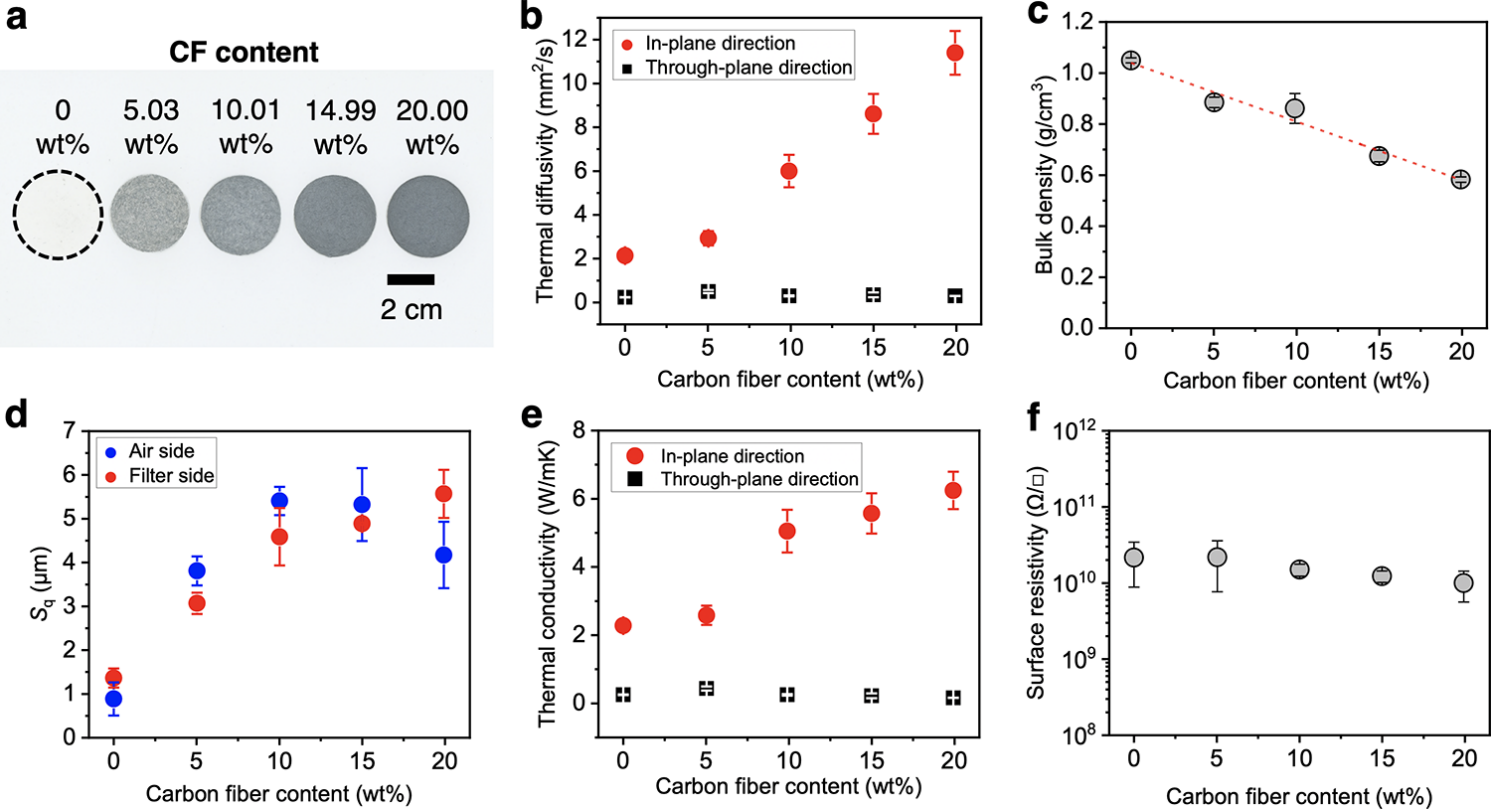
Characterization of the random CF/CNF composite films. (a) Scanned image of the random CF/CNF films with different CF contents. (b) Thermal diffusivity, (c) bulk density, (d) surface roughness expressed as the *S*_q_ value, (e) thermal conductivity, and (f) surface resistivity with respect to the CF content.

To characterize the random CF/CNF films, the properties were evaluated for different amounts of CFs. As the CF content increased, the composite film became darker (Figure 2a). The thermal diffusivity in the in-plane direction increased in proportion to the increase in the CF content, while no change was observed in the through-plane direction (Figure 2b). The properties in the through-plane direction were the same regardless of the CF content. We believe that both CF and CNF fell sideways parallel to the film surface during filtration.

The bulk density of the film decreased with the increasing CF content (Figure 2c). This was contrary to the expectation that the bulk density, which was calculated using a nominal density of 2.2 g/cm^3^ for CF^40^ and the true density of 1.6 g/cm^3^ for CNF,^41^ would increase in proportion to the amount of CF. It is possible that crossing the rigid CFs increased the excluded volume, increasing the numbers of voids and surface irregularities. We then measured the surface roughness of each film, and we found that the *S*_q_ value tended to increase with the increasing CF content (Figures 2d and S2). We speculate that the internal voids and surface irregularities of the films increased during film formation, resulting in a decrease in the apparent bulk density. In the in-plane direction, the thermal conductivity increased in proportion to the CF content (Figure 2e), showing an isotropic in-plane thermal conductivity of 6.2 W/mK at a 20 wt % CF content.

Because thermally conductive substrate materials for electronic devices are required to exhibit electrical insulation, the surface resistance of each film was evaluated (Figure 2f). The surface resistance was high (on the order of 10^10^ Ω/□) regardless of the CF content, indicating that electrical insulation was maintained. This was similar for Aligned-CF10. It is considered that the CNF coated the CF surface to disperse the CFs in aqueous solution, and the top surface of the film was coated with cellulose even after film formation. The CF/CNF films maintained high electrical insulation because of formation in an aqueous CNF suspension, indicating that the films are promising substrate materials for thermal diffusion in electronics.

**Figure 3:**
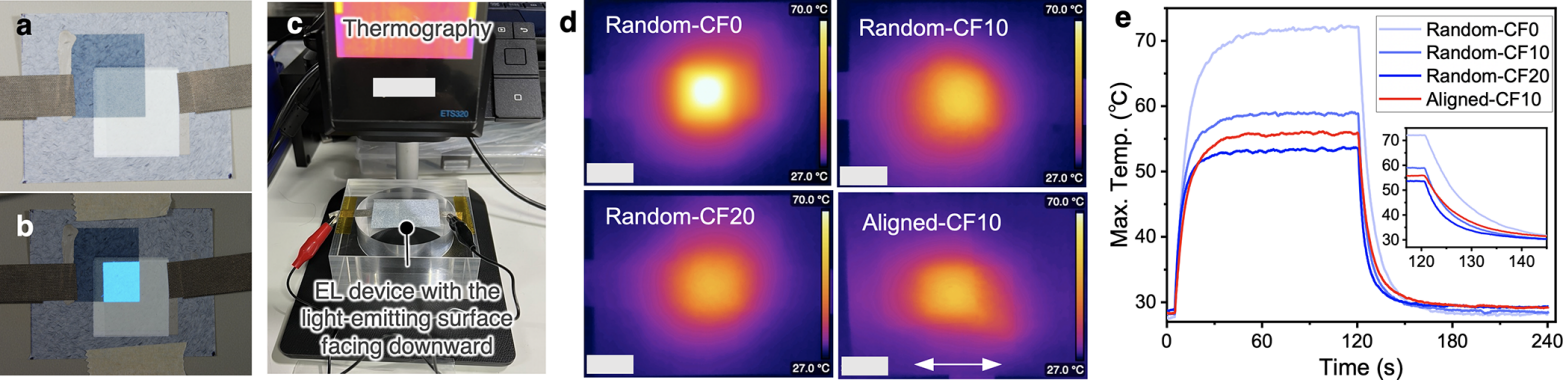
Heat-dissipation tests of the top-emission powder EL devices formed on the CF/CNF films. (a) Appearance of the EL device formed on the Random-CF10 film with dimensions of 4 cm × 5 cm and (b) photograph of the device when emitting light. (c) Setup of the heat-dissipation test for the EL device. (d) Thermographic image of the EL device formed on each substrate ∼105 s after application of an AC voltage of 170 V at 1.2 kHz. The double-headed arrow indicates the direction of CF alignment. (e) Change of the maximum temperature with time. Voltage application was started ∼5 s after the start of the measurement and stopped after 120 s.

Top-emission-type powder EL devices with a 1 cm × 1 cm emission area were formed using the aligned and random CF/CNF films as substrates (Figure 3a), and their heat-dissipation performance was evaluated. Blue surface emission was uniformly obtained when an AC voltage of 170 V at 1.2 kHz was applied to the device (Figure 3b). This indicates that the substrate was electrically insulating and no short circuits occurred, even partially. Additionally, the current and luminance increased with increasing applied voltage (Figure S3), similar to the results of the device with a bottom-emission structure formed using the same phosphor as in the present study.^42^ These results showed that a top-emission-type powder EL device can be driven on the films. Applying an AC voltage of 170 V at 1.2 kHz to the device, thermography images on the back side of the luminescent surface showed that the maximum temperature was significantly different depending on the CF content and alignment. The luminescent area was locally hotter in the Random-CF0 case, but this was mitigated as the CF content increased (Figure 3d). In the Aligned-CF10 case, thermal diffusion in the alignment direction was observed.

AC voltage of 170 V at 1.2 kHz was applied for ∼2 min, the device was left for ∼2 min while the current was stopped, and then the change in the maximum temperature was observed (Figure 3e). The maximum temperature of Random-CF0 increased to ∼72 °C after applying an AC voltage of 170 V at 1.2 kHz for 1 min, while it was suppressed to ∼59 and 53 °C for Random-CF10 and Random-CF20, respectively. The maximum temperature for Aligned-CF10 was ∼56 °C, which was intermediate between the maximum temperatures for Random-CF10 and Random-CF20. Even taking into account that the current and luminance in the powder EL are affected by the unevenness of the substrate surface,^28,42^ the anisotropic substrate is considered to exhibit the same or greater cooling performance than the isotropic substrates with the same CF content. The thermal conductivity and thermal conductivity anisotropy of the substrate were found to clearly control the heat dissipation of the EL device.

**Figure 4:**
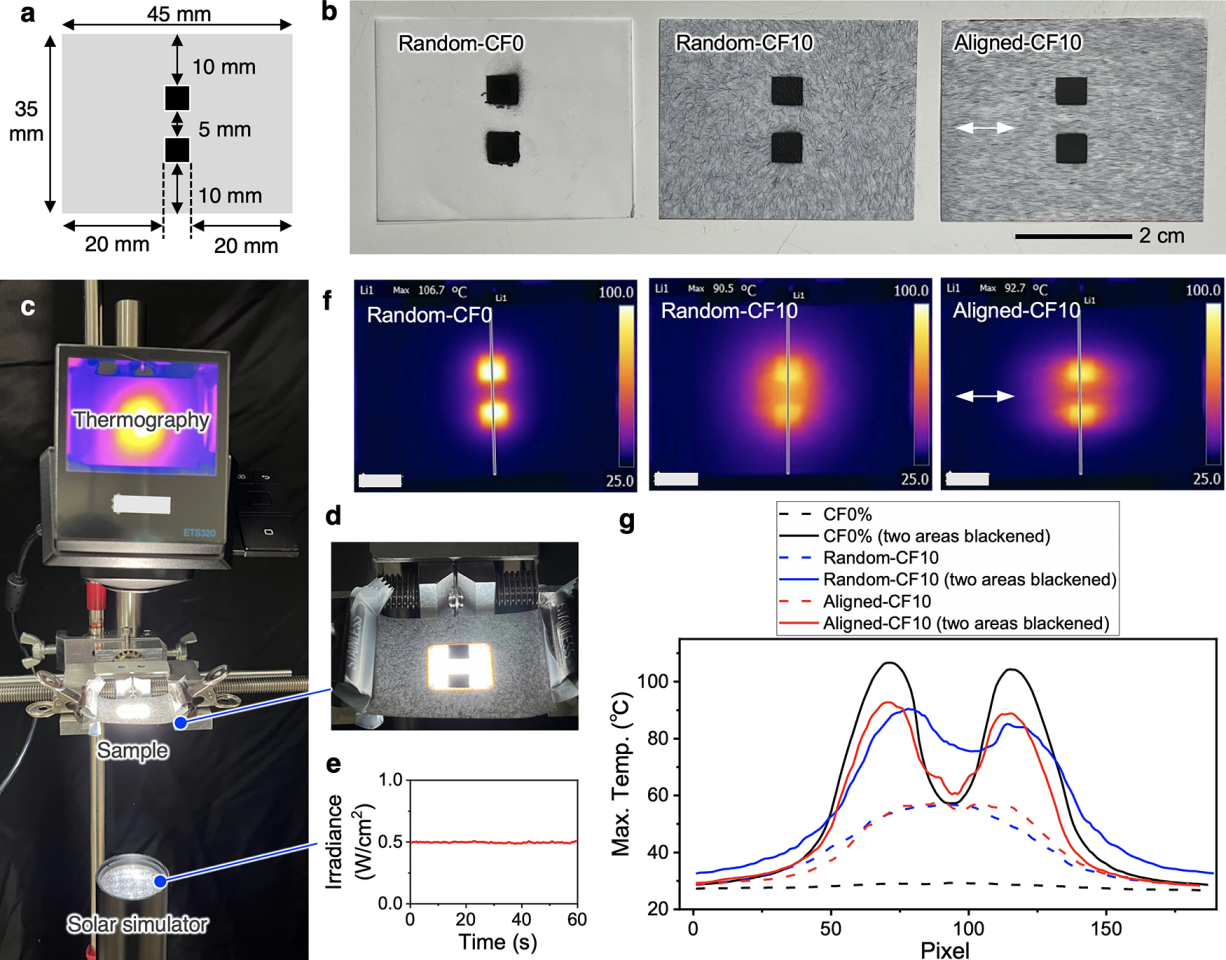
Heat-dissipation tests of two proximate heat sources. (a) Dimensions of the substrate film and placement of the blackened areas as pseudo heat sources. (b) Photograph of the two blackened areas formed on each film. (c) Setup of the heat-dissipation test for the films with two blackened areas. (d) Application of light irradiation to include the two blackened areas. (e) Time profile of the irradiance used. (f) Temperature distribution ∼5 min after the start of light irradiation on each film. (g) Thermographic temperature profiles from line analysis along the straight lines shown in (f) connecting the two ends through the blackened areas.

To investigate the heat-dissipation performance while suppressing the thermal interference between neighboring heating devices, heat-dissipation tests were conducted using two pseudo heat sources. The dimensions of the substrate film were set to 45 mm × 35 mm, and two 5 mm × 5 mm blackened areas were formed in the center as pseudo heat sources, as shown in Figure 4a. For Aligned-CF10, the two blackened areas were placed orthogonal to the CF-alignment direction (Figure 4b). The blackened surface was held on the ground side and irradiated by a solar simulator, and diffusion of the heat generated was observed with a thermal camera (Figure 4c). The area containing the two blackened areas in the center was irradiated (Figure 4d). The target irradiance was set to 0.498 W/cm^2^, which was the energy density when all of the current density (1.66 mA/cm^2^) obtained when 300 V_0-p_ at 1.2 kHz was applied to the powder EL device on the Random-CF0 film was converted to thermal energy. The actual irradiance was ∼0.497 W/cm^2^ on average (Figure 4e).

The two blackened areas on the Random-CF0 film generated localized heat, while the heat on the Random-CF10 film diffused isotropically to the surrounding area (Figure 4f). To clarify the difference in thermal diffusion, we analyzed the temperature distributions between the two ends of the films along the straight lines shown in the thermographic images in Figure 4f. For the Random-CF0 film, the two peaks corresponding to the two blackened areas were prominent, and the temperature increased to ∼105 °C (Figure 4g). The minimum temperature between the blackened areas was ∼57 °C. For the Random-CF10 film, the temperature of the blackened areas decreased to 85–90 °C, while the minimum temperature between the blackened areas increased to ∼76 °C. Thus, isotropic heat-diffusion films remain a significant concern and can cause thermal interference between proximate heat sources.

The temperature of the blackened areas on Aligned-CF10 was 89–93 °C, which was similar to the temperature for Random-CF10 and clearly lower than the temperature for Random-CF0, and the minimum temperature between the blackened areas decreased to ∼60 °C. Considering that Aligned-CF10 had a thermal conductivity of 1.8 W/mK in the alignment orthogonal direction and Random-CF0 had a thermal conductivity of 2.3 W/mK, it is reasonable that the temperatures between the two heat sources were almost the same. Both Random-CF10 and Aligned-CF10 showed a temperature of ∼57 °C by only light irradiation without blackening because CF absorbs light and generates heat. Therefore, the temperature between the blackened areas cannot decrease below 57 °C. Ideally, a measurement system should only generate heat in the two heating areas. However, this is difficult to achieve in heat-dissipation tests using pseudo heat sources with light irradiation. It was technically difficult to form two actual EL devices in proximity on a single substrate. Despite unnecessary heat generation between the two heat sources, Aligned-CF10 clearly reduced both the maximum temperature of the heated area and the temperature between the two heat sources. In other words, the thermally conductive substrate with in-plane anisotropy can simultaneously mitigate localized heat generation and insulate between neighboring heat sources.

As another advantage of combining CFs and CNFs, extraction and the reusability of the CF by combustion treatment were investigated. Preliminary thermogravimetric analysis in air showed that the CF was stable with weight loss of only 2–3% up to ∼600 °C, whereas the CNF lost ∼98% of its weight at ∼385 °C. To establish more detailed temperature conditions for the combustion process, the holding temperature was set to 400, 450, and 500 °C, which are in the intermediate range between the pyrolysis temperatures of cellulose and CF, and thermogravimetric analysis was performed using Random-CF10 to match the temperature increase and decrease rates in the electric furnace (Figure S4). The weight change at the end of the temperature program when the temperature was decreased to ∼42 °C showed an ∼12.5 wt % combustion residue at a holding temperature of 400 °C and an ∼12.3 wt % combustion residue at a holding temperature of 450 °C. There was only an ∼7.99 wt % combustion residue at a holding temperature of 500 °C, indicating that the CF partially decomposed. To extract the 10 wt % CF contained in the film before combustion with the highest purity, it was determined that treatment at 450 °C, where the ash content derived from cellulose was the lowest and CF thermal decomposition did not occur, was suitable.

**Figure 5:**
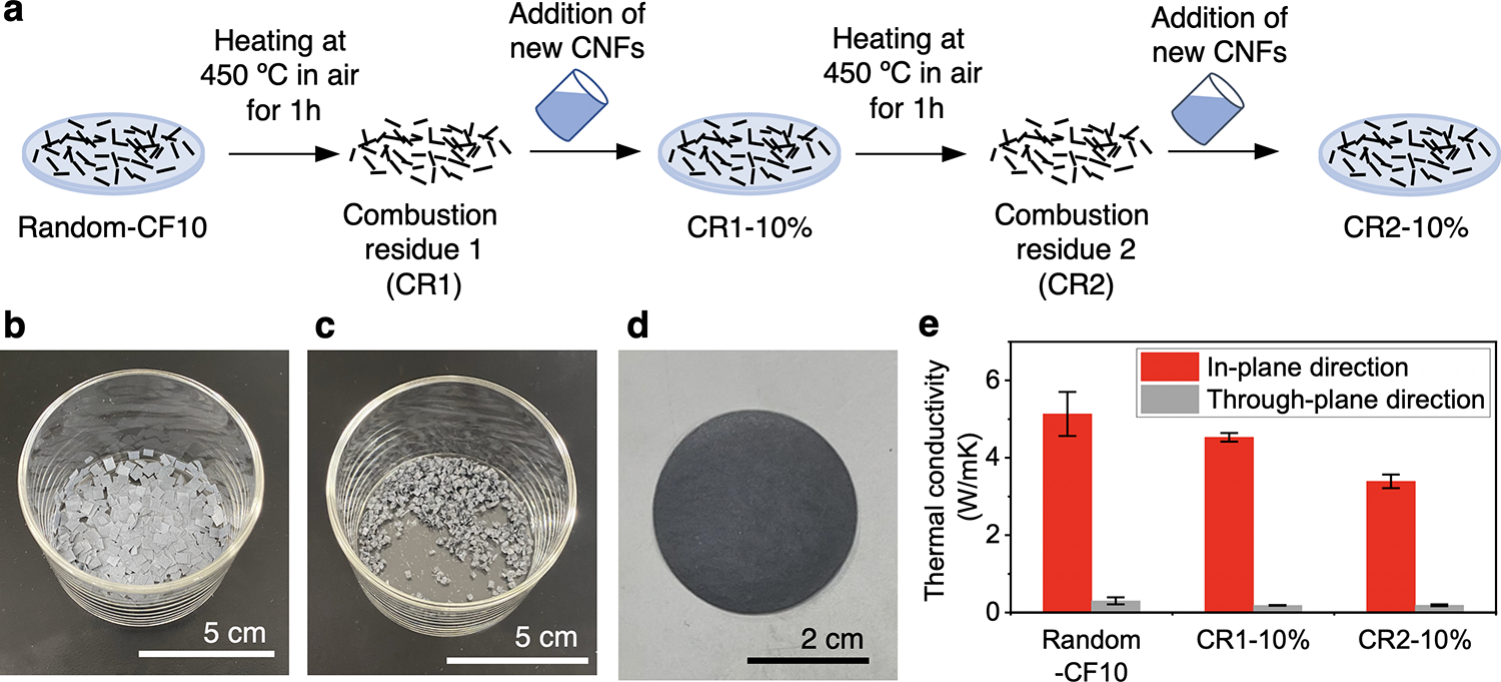
Reusability of the CF filler. (a) Flow of CF extraction by combustion of the CF/CNF film and reuse of CFs as a thermally conductive filler. Appearance of Random-CF10 (b) before combustion and (c) after combustion at 450 °C (i.e., CR1). (d) Photograph of CR1-10%. (e) Thermal conductivity of each film in the in-plane and through-plane directions.

Random-CF10 was used as the starting material (Figure 5a). It was cut into ∼5 mm × 5 mm pieces to avoid uneven combustion (Figure 5b). The residue obtained after 1 h combustion in an electric furnace at 450 °C is designated combustion residue 1 (CR1). After combustion, a mixture of CF and ash was obtained as the residue (Figure 5c). CR1 was added to a new CNF suspension at a 10 wt % filler content to produce random composite film CR1-10%. The new random composite film made from the residue showed no difference in appearance from the film before combustion (Figure 5d). The same combustion and extraction process was then repeated to obtain CR2, followed by formation of the CR2-10% film.

The in-plane thermal conductivity of the films gradually decreased as recycling progressed, from 5.1 W/mK for Random-CF10 to 4.5 W/mK for CR1-10% to 3.4 W/mK for CR2-10%. Of the 12.3 wt % residue obtained from Random-CF10 combustion at 450 °C, ∼2.3 wt % was ash, which made up ∼18.7% of the residue (i.e., the CF purity in CR1 was ∼81.3%). Assuming no thermal decomposition of the ash, the true CF content would decrease to ∼66% in CR2 after two recycling processes, which was consistent with the decrease in the in-plane thermal conductivity. In other words, the combustion process at 450 °C maintained the thermal conductivity of the CF, but the increase in the ash content decreased the true CF content, which lowered the thermal conductivity of the film. However, this result is the first concrete demonstration of the effectiveness of the concept of reusing the filler in thermally conductive composites. It is expected that the thermal conductivity loss can be further reduced by optimizing the temperature program to generate less ash.

## 4 Conclusions

We have fabricated an in-plane anisotropic thermally conductive composite by aqueous mixing of CFs and CNFs and aligning CFs by liquid-phase 3D patterning. We confirmed the in-plane thermal conductivity anisotropy by laser-spot periodic heating radiation thermometry. The aligned film showed a thermal conductivity of 7.8 W/mK in the CF-aligning direction, and it showed the largest in-plane anisotropy of 433% among reported 2D film materials. Powder EL devices formed on this aligned film were significantly cooled by thermal diffusion. Even when two heat sources were mounted, the aligned film simultaneously prevented thermal interference between the heat sources and provided cooling through thermal diffusion in other directions. Further, the CF can be reused as a new thermally conductive filler by extraction from the composite film by heat treatment at 450 °C. These results are considered to be a step toward enabling the design of diverse heat-dissipation patterns for 2D films and using them in a sustainable manner.

## Supporting Information Available

TGA of removal of the CF sizing agent, laser microscopy height profile of each CF/CNF film, voltage dependence of the current density and luminance of the powder EL devices, and TGA of Random-CF10 during CF extraction (PDF)
