# Supplementary material for: Thermal Diffusion Films with In-Plane Anisotropy by Aligning Carbon Fibers in a Cellulose Nanofiber Matrix
Source: ACS Appl Mater Interfaces. 2022 Jul 20;14(29):33903–11. doi: 10.1021/acsami.2c09332 (PMC9335532; doi:10.1021/acsami.2c09332)
Supplement: Supplementary file 1 — am2c09332_si_001.pdf [file am2c09332_si_001.pdf]

Supporting Information

Thermal Diffusion Films with In-Plane Anisotropy by Aligning Carbon Fibers in a Cellulose Nanofiber Matrix

Kojiro Uetani, Kosuke Takahashi, Rikuya Watanabe, Shota Tsuneyasu, Toshifumi Satoh*

*To whom correspondence should be addressed.
E-mail: uetani@ci.tus.ac.jp

This PDF file includes:
Figures S1 to S4

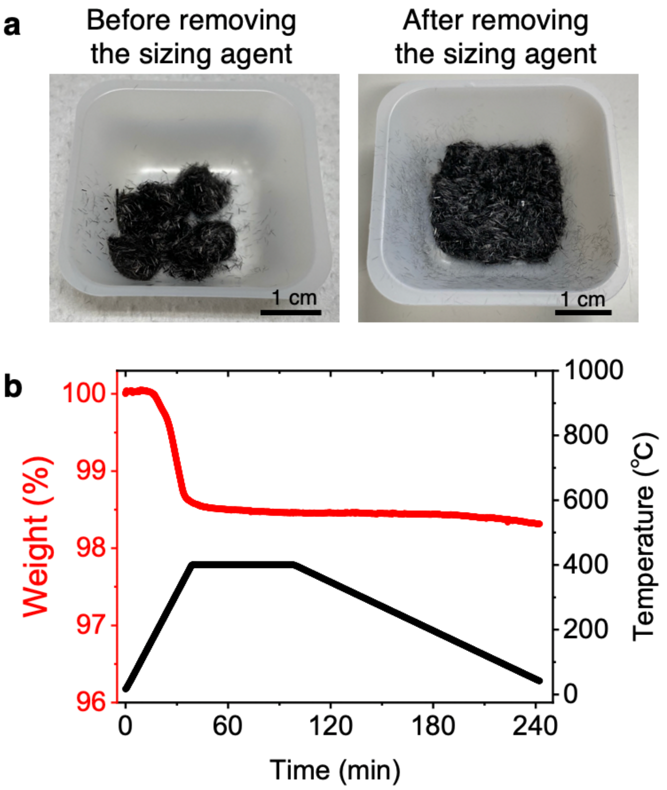

Figure S1. Removal of the CF sizing agent. (a) Appearance of the CFs before and after removing the sizing agent. (b) Weight change of the CFs and the temperature program in the electric furnace.

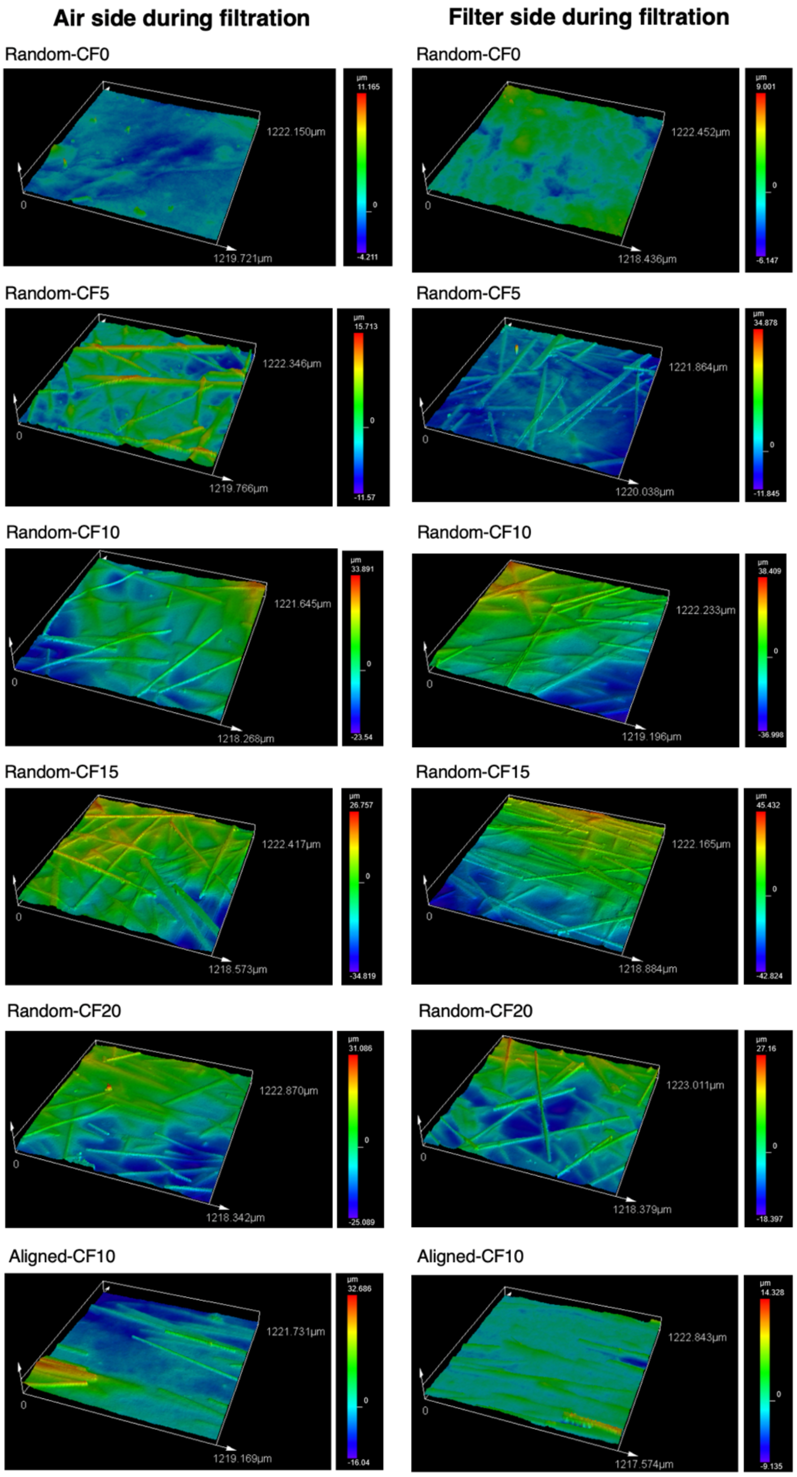

Figure S2. Laser microscopic height-profile images of both sides of each CF/CNF film.

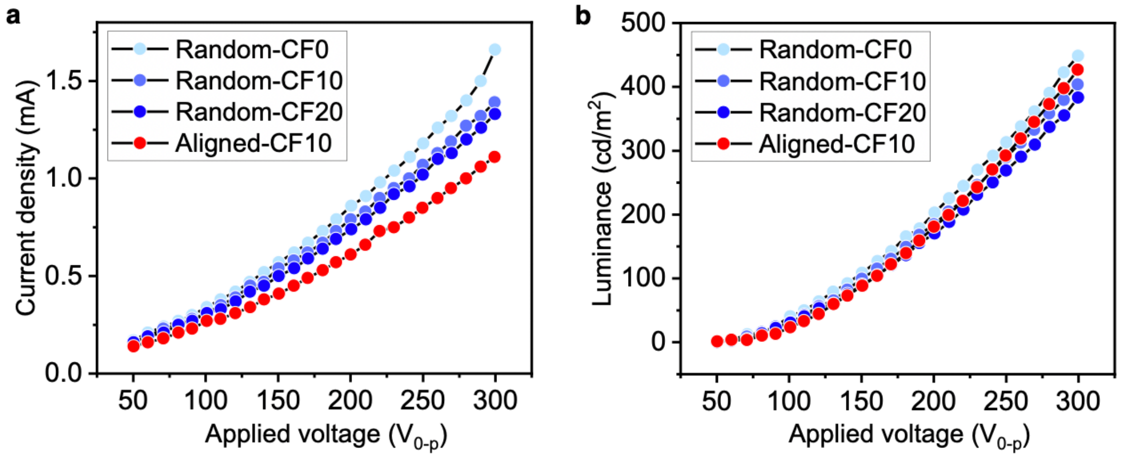

Figure S3. EL characteristics of the top-emission-type powder EL devices based on each CF/CNF film. (a) Current density and (b) luminance versus the applied voltage at an applied frequency of 1.2 kHz.

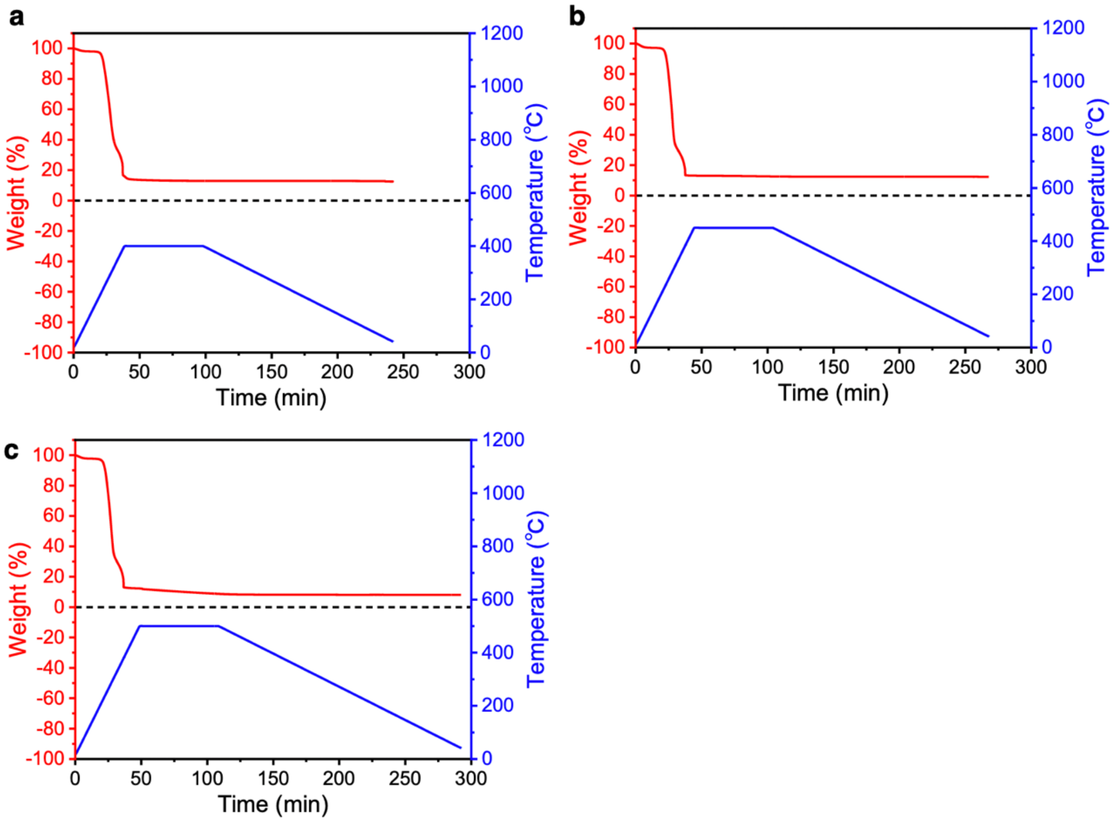

Figure S4. Combustion temperature determination for CF extraction. Weight changes of Random-CF10% in air treated at (a) 400 °C, (b) 450 °C, and (c) 500 °C for 1 h, along with the temperature programs.
